# A novel spectrophotometric approach relies on a charge transfer complex between atomoxetine with quinone-based π-acceptor. Application to content uniformity test

**DOI:** 10.1186/s13065-023-00951-0

**Published:** 2023-04-22

**Authors:** Ahmed A. Abu-hassan

**Affiliations:** Department of Pharmaceutical Analytical Chemistry, Faculty of Pharmacy, Al-Azhar University, Assiut branch, Assiut, 71524 Egypt

**Keywords:** Atomoxetine, Complexe, p-chloranil, Spectrophotometric, DDQ

## Abstract

Atomoxetine (ATO) belongs to psychoanaleptic drugs and is utilized in attention-deficit hyperactivity syndrome treatment. In this study, two facile and selective approaches are implemented for the spectrophotometric analysis of atomoxetine. The two approaches rely on charge transfer formed between ATO base (n-donor) with p-chloranil and 2,3-dichloro-5,6-dicyano-p-benzoquinone (DDQ; π-acceptor). The generated complexes exhibit absorption intensity maxima at 550 and 460 nm in acetonitrile for p-chloranil and DDQ in the order. Under the optimum reaction condition, Beer’s law was followed for p-chloranil and DDQ at concentrations of 30–320 and 10–80 µg mL^− 1^, respectively. The ICH guidelines were followed for work validation, and the outcomes were excellent and satisfactory. The assessment of ATO in pharmaceutical capsules using the suggested procedures was successful, and the results were contrasted with those obtained using a different published method to show accuracy and precision. Additionally, the two methods were used to assess the homogeneity of ATO content in the commercialized capsule.

## Introduction

**Fig. 1:**
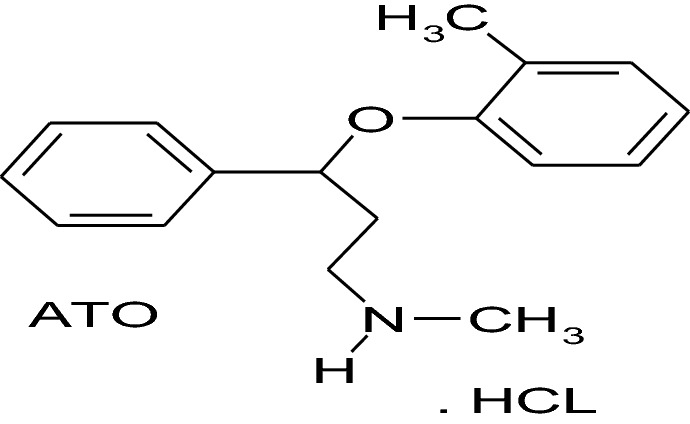
Chemical structure of atomoxetine

 Atomoxetine HCl has the chemical name (R) n-methyl-3-(2-methylphenoxy)-3-phenyl propylamine hydrochloride, and M.Wt of 291.8 (Fig. 1). The stated medication is used to treat attention deficit hyperactivity syndrome. The most prevalent neurobehavioral condition of childhood is ADHD, and some individuals’ symptoms last into adulthood. It is characterised by signs of impulsivity, hyperactivity, and inattentiveness that interfere with academic and social performance [1, 2].

Several strategies have been reported for ATO assay. Among these methods are spectrophotometry [3–5], spectrofluorimetry [2, 6], liquid chromatography [7–10], capillary electrophoresis [11] and electrochemical method [12].

The suggested techniques perform measurements in the visible region(colorimetry), away from UV-absorbing interfering excipients that might be co-extracted from the dosage form containing ATO, making them superior to UV-based spectrophotometric techniques. The suggested procedures also make use of inexpensive reagents and a spectrophotometer, both of which are common in quality control laboratories.

The production of vibrantly colored charge-transfer (ATO-π acceptor) complexes, which absorb light in the visible spectrum, is typically linked to the molecular interactions between ATO (electron donors) and electron π acceptors. Numerous chemicals that donate electrons have been found to produce charge-transfer complexes, which makes them useful for the creation of straightforward and practical spectrophotometric techniques [13–16]. In this study, we introduce novel spectrophotometric techniques that rely on the interaction between the secondary amine moiety of ATO acting as the n-donor and p-chloranil and DDQ acting as the -acceptors. The proposed methods’ selectivity, sensitivity, and precision, as well as their application to pharmaceutical capsules, were investigated. Complex affecting variables were thoroughly studied and controlled. The strategy was employed in checking capsule uniformity and homogeneity for the first time.

## Experimental

### Apparatus

A quartz cell spectrophotometer with a 1 cm optical path length made by Shimadzu, model UV-1601, was employed. An electronic single pan balance (Precisa XB 220 A, Switzerland) was used for all weighing operations. A water distiller prepared the distilled water (TYUMEN-MIDI-A0-25 MO, Russia).

### Chemical and reagents

Analytical reagents were all employed without further purification. Mash Premiere for Pharmaceutical Industries (Badr city, Egypt) provided pharmaceutical grade atomoxetine hydrochloride and p-chloranil (Sigma Chemical Co., USA) 200 mg of p-chloranil were dissolved in a 100 mL volumetric flask with acetonitrile as the solvent to create 0.2% w/v. 2,3-dichloro-5,6-dicyano-p-benzoquinone (DDQ) (Sigma Chemical Co., USA) 0.3% w/v is produced by combining 300 mg of DDQ with acetonitrile and dissolving it in a 100 mL standard flask. El Nasr Chemical Co. provided the ethanol, methanol, acetonitrile, 1,4-dioxane, and dimethylformamide that were purchased (Abu Zaabal, Egypt).

### Pharmaceutical formulation

Atomox apex capsules (Multi-Apex Pharma, Cairo, Egypt) labeled to contain 25 mg and Strattera 18 mg ATO per capsule, were purchased from the local market.

### Stock standard solutions for chloranil and DDQ methods

Aqueous ammonia Solution 33–34% is used to make the solution alkaline after the correctly measured 200 mg of atomoxetine hydrochloride has been dissolved in 40 mL of distilled water. Next, 20 mL of diethyl ether [17] is added to the flask. The preceding aqueous layer is emptied back into the flask after the two phases have been agitated for 10 min and separated after that. Two further extractions of the aqueous phase using 20 mL of diethyl ether are performed. Each time, a 100 mL separating funnel containing the organic layer was filtered through sodium sulphate, and the filter paper was rinsed with 20 mL of diethyl ether. The free base was recovered with acetonitrile after the organic phase was evaporated at 40–50 °C.

### General analytical procedures

A series of 10-mL (calibrated) flasks were filled with aliquot volumes of standard stock solutions comprising 100–3200 µg mL^− 1^ of medication per ml. 1 mL of DDQ (0.3% w/v) or 0.8 mL of p-chloranil (0.2% w/v) were each added and diluted with acetonitrile to the desired concentration. After 5 min, the absorbance of the resultant solutions was compared to reagent blanks prepared identically at wavelengths of maximum absorption of 460 and 550 nm for DDQ and p-chloranil, respectively.

### Analysis of ATO in capsules and checking of content uniformity

Twenty capsules worth of substance were emptied, precisely weighed, and thoroughly blended. 200 mg of the medication salt equivalent in mixed capsule form was precisely weighed and transferred to a volumetric flask measuring 100 mL. The first part of the filtrate was dumped after the solution was filtered. Using the same technique used to make a standard stock solution, 100 mg of the filtrate was converted to a free base. Applying the usual analytical approach allowed us to determine the drug content of the final solutions. The same procedures were used to assess the Strattera 18 mg capsules for content homogeneity, however, each capsule was examined independently and reconstituted with acetonitrile in a 25 mL volumetric flask.

### Molar ratio study

By creating master solutions of the drug and -acceptor with the same molar concentration, Job’s approach of continuous variation [18] was used (0.0068 M) of both. A series of solutions were created with a total volume of 1 mL for ATO and π-acceptor. The chemicals were combined in a variety of ratios, diluted with acetonitrile to volume in a 10-mL calibrated flask, and the absorbance was tested at max against an identically created blank solution.

## Result and discussion

With DDQ and p-chloranil in acetonitrile, ATO produces vibrant colours with maximum absorption at wavelengths of 460 and 550 nm, respectively. This is because DDQ or p-chloranil, acting as Lewis acid, and ATO, acting as n-donor or Lewis base, produce charge-transfer complexes


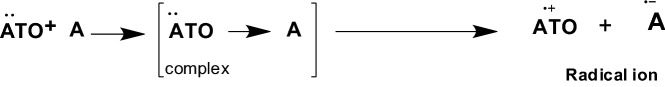


Acetonitrile was regarded as the optimal solvent since it provided the highest sensitivity and the highest production of radical anions due to its strong dielectric constant [15] and significant reagent solvating ability.

### Absorption spectrum

**Fig. 2:**
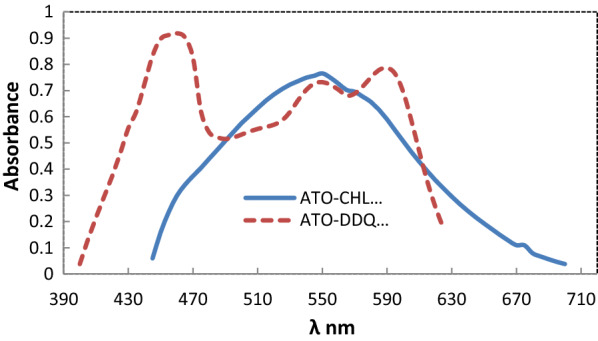
Absorption spectra of charge transfer complex using 70 and 280 µg mL^− 1^ for chloranil and DDQ respectively

**Scheme 1:**
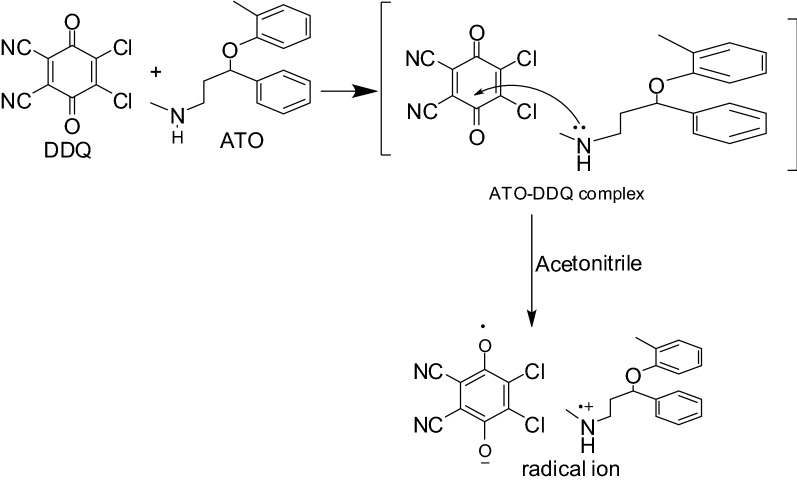
Suggested mechanism of reaction of ATO and DDQ as representative example

Using a spectrophotometer, the absorption spectra of charge transfer complexes produced by the reaction of p-chloranil or DDQ (π-acceptor) and ATO (n-donor) were surveyed over the wavelength range of 200–800 nm. It was discovered that the produced chromogen shows maximum absorption for DDQ and p-chloranil at 460 and 550 nm, respectively (Fig. 2). The creation of ion radicals as a result of the original complex’s dissociation is attributed to these bands. The high ionization power of acetonitrile facilitates dissociation [19] (Scheme 1).

### Adjusting reaction conditions

The stability of the colored product, reaction time, and effect of solvent dilution were among the experimental factors that were optimized at room temperature (25 °C). To do this, each parameter was changed while the values of the others were left unchanged, and the effects on the product’s absorbance peak were tracked.

#### Impact of reagent concentration

**Fig. 3:**
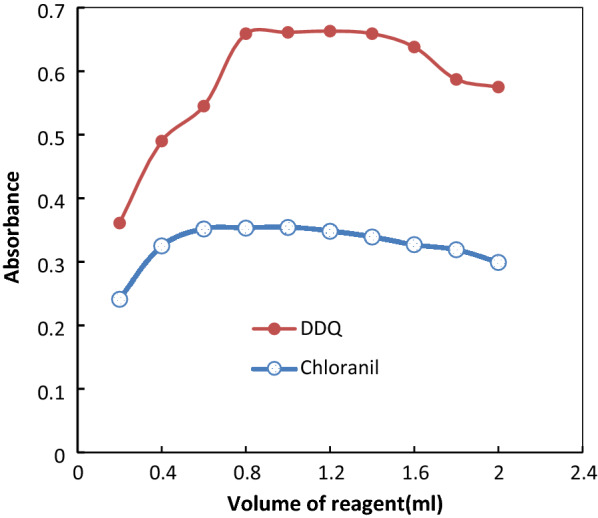
Effect of volume of chloranil 0.2% w/v and DDQ 0.3% w/v on absorbance of color product with ATO 50 and 120 µg mL^− 1^ for DDQ and chloranil respectively

By altering the volume of a constant concentration, the impact of the reagent concentration on the intensity of the colour created was tested. The ideal concentration was found to be 1 mL of 0.3% w/v DDQ and 0.8 mL of 0.2% w/v chloranil (Fig. 3).

#### Impact of time on color product development and stability

**Fig. 4:**
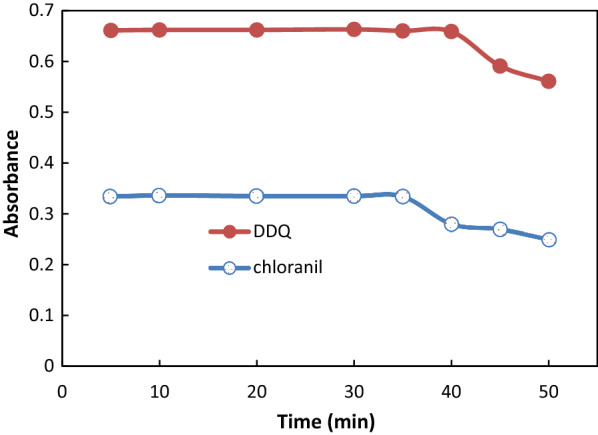
Effect of time on absorbance of reaction product obtained from reaction of ATO 50 µg mL^− 1^ with DDQ and 120 µg mL^− 1^with chloranil

Monitoring the color development at ambient temperature allowed researchers to identify the ideal response time. After combining the solutions, full-color development was achieved instantly. For a more accurate result, measurements were taken after 5 min. For the DDQ and chloranil procedures, the generated colors were stable at room temperature for 35 and 40 min, respectively (Fig. 4).

#### Diluting solvent impact

**Fig. 5:**
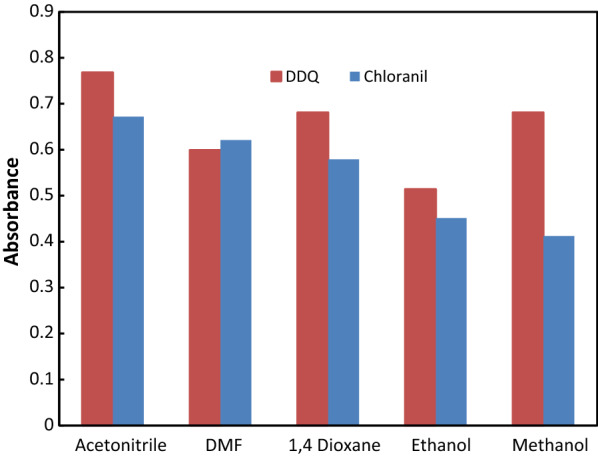
Effect of different diluting solvent on absorbance of reaction product obtained from reaction of ATO 60 µg mL^− 1^ with DDQ and 240 µg mL^− 1^with chloranil

For choosing the best solvent, some different solvents, including acetonitrile, methanol, ethanol, 1,4-Dioxane, and dimethylformamide, were examined. The two-acceptors were thought to respond best to acetonitrile as a solvent. This is because it provided the highest sensitivity, which was linked to the high dielectric constant of acetonitrile, which fosters the highest yield of radical anions, as well as its high solvating strength for acceptors (Fig. 5).

**Fig. 6:**
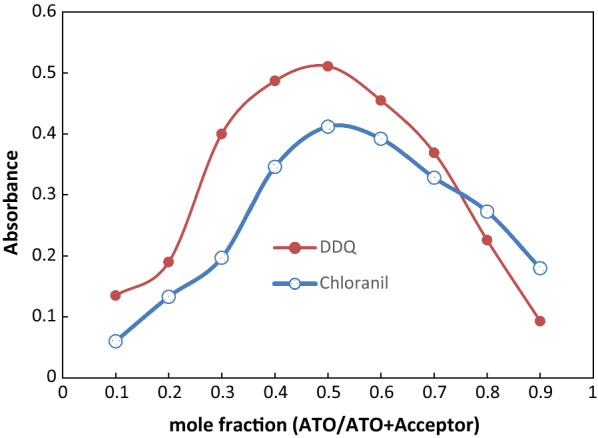
Jobs plots for molar ratio determination using equi-molar solutions (0.0068 M) of both ATO and π-acceptors at optimum wave length

#### The stoichiometry of the reaction

Using Job’s approach, the stoichiometry of the interactions was investigated. The ratio between the donor (ATO) and acceptors (chloranil and DDQ) was discovered to be 1:1 (Fig. 6).

### Method validation

After the approach was created, it was validated in accordance with the standards established by the International Conference on Harmonization (ICH) [20]. The validation criteria included robustness, accuracy, linearity, and detection and quantitation limitations.

#### Linearity and range

**Fig. 7:**
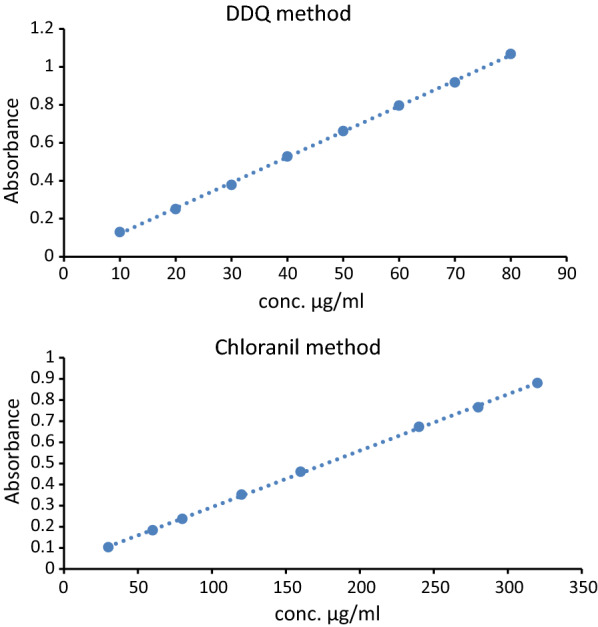
The calibration curve of the proposed methods

By using the suggested procedures under ideal reaction circumstances, a number of typical medication solutions with varying concentrations were examined. Plotting the absorbance (A) vs. the matching final medication concentrations resulted in the calibration graph. The least squares method was used to create the regression equations for the outcome. The Beer’s law plots for the three methodologies (chloranil and DDQ) were linear with extremely tiny intercepts and strong correlation coefficients (0.9998 and 0.9997) in the concentration ranges of 30–320 and 10–80 µg mL^− 1^, respectively. Table 1 contains the estimated analytical variables and statistical information. Figure 7 shows the calibration curves for both methods.

**Table 1:** Summary of quantitative parameters and statistical data using the proposed methods

Parameters	Chloranil method	DDQ method
Linear range (µg mL^− 1^)	30–320	10–80
Intercept (a)	0.0259	− 0.0134
Standard deviation of intercept (SD _a_)	0.004007	0.00637
Slop (b)	0.0027	0.0134
Standard deviation of slope (SD _b_)	0.000021	0.00013
Correlation coefficient (r)	0.9998	0.9997
Determination coefficient (r ^2^ )	0.9996	0.9995
Molar absorptivity Ɛ (L / mol cm )	858.64	3805.107
Limit of quantitation (LOQ), µg mL ^− 1^	14.84	4.75
Limit of detection (LOD), µg mL ^− 1^	4.89	1.56

Number of determinations were 8 for both methods

#### Quantitation and detection limits

The detection (LOD) and quantitation (LOQ) limits were calculated to determine the proposed strategy’s sensitivity. The computation was based on the calibration curve’s slope and the standard deviation of intercept. The results are reported in Table 1 using the calculations LOD = 3.3σ/S and LOQ = 10σ/S.

#### Accuracy

For the study of five reference ATO solutions with varying concentrations inside the advised range, the usual analytical approach was used. There were three replicate measurements made for every concentration level. Table 2’s data were presented as recovery%. The calculated recovery percentage’s proximity to 100% provides a clue as to the method’s tolerable accuracy.

**Table 2:** Evaluation of accuracy of the proposed spectrophotometric methods

Chloranil method			DDQ method		
Taken µg mL^− 1^	found µg mL^− 1^	%Recovery^a^	Taken µg mL^− 1^	Found µg mL^− 1^	%Recovery^a^
60	59.05	98.42	20	19.78	98.91
80	78.68	98.35	30	29.43	98.11
120	119.30	99.41	40	39.43	98.58
160	160.16	100.10	60	59.86	99.76
240	242.51	101.04	70	69.23	98.91
Mean		99.46	Mean		98.85
SD		1.15	SD		0.60

^a^The value is the average of three determinations, SD is the standard deviation

#### Precision

The repeatability and intermediate precision of the suggested approach were both examined. The study was run three times in a row on the same day to obtain repeatability (intra-day precision). In the instance of the intermediate (inter-day) precision, the analysis was performed over three consecutive days. The result of the calculation to determine the relative standard deviation (RSD) is reported in Table 3. The low value of % RSD, which did not surpass 2%, served as evidence of the proposed methodologies’ high degree of precision.

**Table 3:** Evaluation of intra-day and inter-day precision for the determination of atomoxetine by the proposed analytical procedures

Precision level	Chloranil method			DDQ method		
	Conc. (µg mL^− 1^)	%Recovery^a^ ± SD	% RSD	Conc. (µg mL^− 1^)	%Recovery^a^ ± SD	% RSD
Intra-day	80	98.50 ± 0.71	0.72	30	98.11 ± 0.75	0.76
	120	100.75 ± 0.47	0.47	40	101.19 ± 0.19	0.18
	160	99.95 ± 0.35	0.35	60	100.30 ± 0.33	0.33
Inter-day	80	98.04 ± 0.71	0.72	30	98.69 ± 0.57	0.58
	120	101.37 ± 1.17	1.15	40	100.88 ± 0.39	0.38
	160	98.94 ± 0.94	0.95	60	98.85 ± 1.21	1.22

^a^ The value is the mean of three determinations; SD and RSD are the standard deviation and relative standard deviation, respectively

#### Robustness

In carrying out the overall analytical method, minor adjustments were made to the experimental variables (reagent volume, stability time). Calculating % recovery and SD at each iteration allowed researchers to assess how these modifications affected the analytical performance of the approach (Table 4). The outcomes demonstrated that these modifications had no discernible impact on the analysis, demonstrating the robustness of the established spectrophotometric approach. This demonstrates the suggested method’s dependability in a typical application.

**Table 4:** Robustness of the proposed methods for determination of atomoxetine using 120 and 60 µg mL^− 1^ for chloranil and DDQ respectively

Parameters	Value	Chloranil method	DDQ method
		% Recovery^a^ ± SD	% Recovery^a^ ± SD
Reagent volume(mL)	+ 0.2	101.16 ± 1.46	100.22 ± 0.40
	−0.2	99.21 ± 1.70	98.76 ± 0.19
Stability time(min)	15	99.11 ± 1.05	98.89 ± 1.7
	25	98.03 ± 1.52	98.33 ± 0.98

^a^The mean value of three determinations, SD is the standard deviation

**Table 5:** Assessment of the selectivity for the suggested methods in presence of some common excipients

Excipients	Amount added (µg mL^− 1^)	Chloranil method	DDQ method
		Drug con. (120 µg mL^− 1^)	Drug con. (40 µg mL^− 1^)
		% Recovery ± SD^a^	% Recovery ± SD^a^
Mg stearate	15	101.16 ± 1.46	100.45 ± 1.13
Starch	50	99.21 ± 1.70	99.14 ± 1.80
Lactose	15	100.96 ± 1.60	98.02 ± 0.19
Glucose	15	98.28 ± 1.08	100.88 ± 0.39
Talc	15	99.41 ± 1.35	98.02 ± 0.49
Sorbitol	15	98.49 ± 0.93	101.13 ± 0.11

#### Selectivity

The effects of each excipient were examined separately to make sure there was no variation from the common excipients. Glucose, starch, talc, lactose, sorbitol, and magnesium stearate were among the excipients that were examined. The estimated results shown in (Table 5) demonstrate that the excipient did not have a significant effect on the final result. The amino group, which is missing in the typical excipient, is what gives the method its selectivity.

## Strategy application in capsules and uniformity inspection

The recommended techniques had been used in turn to determine the presence of atomoxetine in Atomox Apex capsules. By using the student’s t-test and F-test, respectively, the findings produced by the proposed approaches were statistically compared with those obtained by the stated method [21] regarding accuracy and precision. It was discovered that the estimated values for both tests fell within the 95% confidence interval of the tabulated value (Table 6). This affirms the suggested method’s excellent accuracy and precision. Furthermore, the recovery rates attained by the current method were very near to 100%, indicating that the inclusion of the frequently used excipients in the dosage forms under investigation did not affect the method’s outcomes. As a result, the suggested method can be used in quality control laboratories to analyze the dose forms of the pharmaceuticals mentioned. The approaches that were offered were expanded to test the homogeneity of the content of Strattera 18 mg. The fact that the anticipated AV value was less than the maximum allowed value demonstrates the formula’s excellent consistency with ATO (Table 7). Finally, a comparison of the current work with some reported spectrophotometric methods is outlined in Table 8.

**Table 6:** Analysis of atomoxetine in pharmaceutical dosage form using the proposed and reported methods [21]

Dosage form	Chloranil method	DDQ method	Reported method
	% Recovery ^a^ ± SD	% Recovery ^a^ ± SD	% Recovery ^a^ ± SD
Atomox APEX capsules	98.92 ± 1.85	98.96 ± 1.59	100.63 ± 1.19
t-value ^b^	1.74	1.88	
F- value ^b^	2.42	1.78	

^a^ The value is the average of five determinations for both the proposed and reported methods

^b^ Tabulated values at 95% confidence limit are t = 2.306, F = 6.338

**Table 7:** Content uniformity checking of Strattera 18 mg by the current methods

Capsule number	Chloranil method	DDQ method
1	102.91	98.76
2	99.21	100.22
3	101.16	98.76
4	98.28	100.22
5	101.16	98.76
6	97.56	97.85
7	96.74	97.60
8	99.21	100.22
9	101.16	98.31
10	99.21	99.28
Mean ($\overline{X}$) ± S.D	99.66 ± 1.91	1.38
Acceptance value (AV)	4.58	3.30
Max. allowed AV (L1)	15	15

**Table 8:** Comparison of the current work with some reported spectrophotometric methods

Items	Proposed method	Reported method [3]	Reported method [4]	Reported method [5]	Reported method [6]
λ_max_	550 and 460 nm for method I and II	560 and 600 nm for method I and II	520.5 and 450.6 nm for method I and II	550 nm	449 nm
Reagent	p-chloranil and 2,3-dichloro-5,6-dicyano-p-benzoquinone	Vanillin or Para dimethyl amino Benzaldehyde	Sodium meta periodate and Folin reagent	Gold (III) chloride	1,2-naphthoquinone-4-sulphonate
Linear range	30–320 and 10–80 µg mL ^− 1^	1–5 µg/mL for method I and 10–50 µg/mL for method II	(4.0–20) µg/mL for method I, (16–48) µg/mL for method II	5–80 µg/mL	5–40 µg mL ^− 1^
LOQ (µg mL ^−1^ )	14.84 and 4.75	Not reported	Not reported	7.6170 and 34.7446	0.06
LOQ (µg mL ^−1^ )	4.89 and 1.56	Not reported	Not reported	2.2837 and 10.3723	0.02
Application	Capsules and content uniformity testing	Capsules	Capsules	Tablets	Capsules

## Conclusions

Atomoxetine as an electron donor and two electron acceptors have been studied in the charge-transfer complexation reaction. Visible spectrophotometry was used to investigate the produced complexes. The developed spectrophotometric methods for ATO analysis in pure forms, capsules, and checking content homogeneity made use of the colored complexes that were produced. The suggested techniques perform measurements in the visible region(colorimetry), away from UV-absorbing interfering excipients that might be co-extracted from the dosage form containing ATO, making them superior to UV-based spectrophotometric techniques. The suggested procedures also make use of inexpensive reagents and a spectrophotometer, both of which are common in quality control laboratories.

## Data Availability

The data will be available upon reasonable request from the corresponding author.
